# Ligand Binding Affinities of Arctigenin and Its Demethylated Metabolites to Estrogen Receptor Alpha

**DOI:** 10.3390/molecules18011122

**Published:** 2013-01-16

**Authors:** Jong-Sik Jin, Jong-Hyun Lee, Masao Hattori

**Affiliations:** 1 Institute of Natural Medicine, University of Toyama, 2630 Sugitani, Toyama 930-0194, Japan; 2 College of Pharmacy, Dongduk Women’s University, 23-1 Wolgok-Dong, Sungbuk-Gu, Seoul 136-714, Korea

**Keywords:** arctigenin, estrogen receptor alpha, demethylation, ligand binding affinity

## Abstract

Phytoestrogens are defined as plant-derived compounds with estrogen-like activities according to their chemical structures and activities. Plant lignans are generally categorized as phytoestrogens. It was reported that (−)-arctigenin, the aglycone of arctiin, was demethylated to (−)-dihydroxyenterolactone (DHENL) by *Eubacterium (E.)* sp. ARC-2. Through stepwise demethylation, *E.* sp. ARC-2 produced six intermediates, three mono-desmethylarctigenins and three di-desmethylarctigenins. In the present study, ligand binding affinities of (−)-arctigenin and its seven metabolites, including DHENL, were investigated for an estrogen receptor alpha, and found that demethylated metabolites had stronger binding affinities than (−)-arctigenin using a ligand binding screen assay method. The IC_50_ value of (2*R*,3*R*)-2-(4-hydroxy-3-methoxybenzyl)-3-(3,4-dihydroxybenzyl)-butyrolactone was 7.9 × 10^−4^ M.

## 1. Introduction

Some plant lignans have been categorized as phytoestrogens or their precursors with isoflavones because natural compounds and/or their metabolites act like estrogen [1,2,3]. Estrogenic and anti-estrogenic activities of phytoestrogens have been studied for a long time and potential hormone-like effects were assumed [4,5,6]. The dietary lignans such as secoisolariciresinol diglucoside, pinoresinol diglucoside, sesamin, asarinin, tracheloside and arctiin are transformed to mammalian lignans, enterolactone (ENL) and enterodiol (END) by human intestinal bacteria [3,7,8,9,10,11]. Many human intestinal bacteria having metabolic activities are involved in the biotransformation [7,8,12,13,14,15,16,17,18,19,20]. As a result, the bacterial metabolism not only produces END and ENL, mammalian lignans, but also diverse metabolites.

Six metabolites are produced by incubation of arctiin, abundant in the seeds of *Arctium *(*A.*) *lappa*, with human intestinal bacteria [10]. The metabolic processes include deglucosylation, demethylation, and dehydroxylation. Since arctiin has three methoxy groups in aromatic rings and demethylation occurs prior to dehydroxylation [8], this showed that the demethylation process is quite important for producing enterolactone.

**Figure 1:**
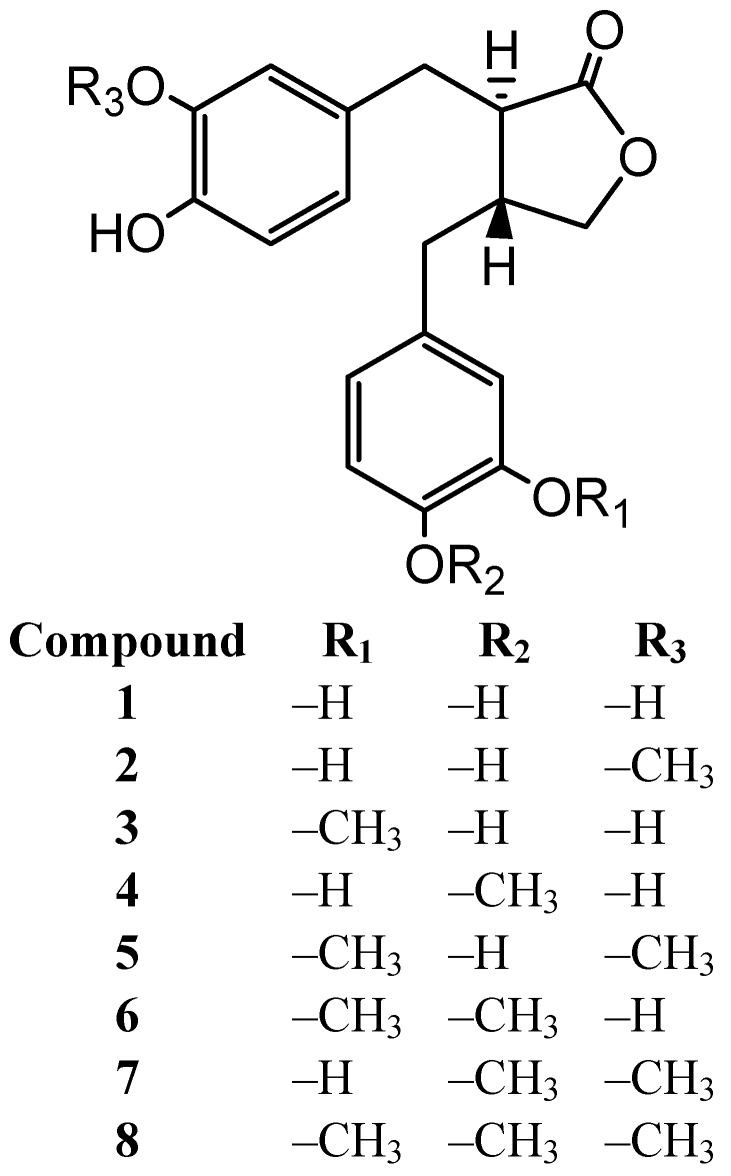
Arctigenin and its metabolites by *E.* sp. ARC-2.

*Eubacterium* (*E.*) *limosum* ARC-2 is isolated as a human intestinal bacterium, capable of demethylating arctigenin from human feces [20]. (−)-Acrtigenin (**8**) is metabolized to (−)-dihydroxyenterolactone (DHENL, **1**) through stepwise demethylation. Six intermediates **2**–**7** are isolated, as shown in the previous paper (Figure 1).

In present study, we examined the binding affinity of (−)-arctigenin (**8**) and its metabolites **1**–**7** using an estrogen receptor alpha (ERalpha) competitor screening assay.

## 2. Results and Discussion

The half maximal inhibitory concentration (IC_50_) value of (−)-arctigenin (**8**) was higher than 5 × 10^−3^M (Table 1). Out of three mono-desmethylarctigenins **5**–**7**, including (−)-matairesinol (**5**), the IC_50_ value of metabolite **6** was 2.5 × 10^−3^ M, being lower that that of the original compound **8**. This finding agreed with a previous observation by Xie *et al.*, who showed metabolite **6** stimulated the proliferation of MCF-7 human breast cancer cells, having estradiol-mediated proliferative characteristics [10]. Metabolite **6** is a main metabolite in incubation of arctiin with human and rat intestinal flora *in vitro* [10,21]. However, when arctigenin (**8**) was incubated with *E*. *limosum* ARC-2, the major mono-desmethylarctigenin was metabolite **7** [20]. It means that the composition of human intestinal microbiota could change the metabolic pattern of arctigenin (**8**), followed by alteration of estrogenic activity.

**Table 1:** Ligand binding affinities of arctigenin and its metabolites to ER alpha.

Compound	% Binding to ERα				IC_50_	RBA
	10^−5^ M	10^−4^ M	5 × 10^−4^ M	5 × 10^−3^ M		
**1**	5.6 ± 3.3 *	13.7 ± 0.7 **	34.8 ± 1.3 **	65.3 ± 0.2 **	1.6 × 10^−3^ M	1.2 × 10^−3^
**2**	1.4 ± 1.2	13.5 ± 2.0 **	44.4 ± 0.9 **	67.7 ± 0.2 **	7.9 × 10^−4^ M	2.5 × 10^−3^
**3**	2.2 ± 1.6	14.1 ± 1.3 **	25.8 ± 0.8 **	67.5 ± 0.2 **	1.9 × 10^−3^ M	1.0 × 10^−3^
**4**	1.0 ± 1.4	−0.5 ± 0.7	19.6 ± 2.3 **	63.2 ± 0.3 **	2.5 × 10^−3^ M	7.9 × 10^−4^
**5**	0.4 ± 0.9	18.1 ± 9.0 **	0.1 ± 2.6	17.6 ± 1.1 **	>5 × 10^−3^ M	–
**6**	−2.3 ± 0.9	1.4 ± 2.4	16.6 ± 0.9 **	64.5 ± 0.2 **	2.5 × 10^−3^ M	7.9 × 10^−4^
**7**	3.6 ± 0.6	1.2 ± 1.7	−4.2 ± 0.8	30.1 ± 1.7 **	>5 × 10^−3^ M	–
**8**	−1.4 ± 0.8	−4.3 ± 0.8	−4.1 ± 3.5	19.4 ± 1.5 **	>5 × 10^−3^ M	–

Estradiol IC_50_ = 1.99 × 10^−8^ M. RBA (Relative binding affinity) was calculated by dividing the IC_50_ of estradiol by that of the tested compound and multiplied by 100, Estradiol (RBA = 100). Asterisks denotes significant difference from the control at (*) *p* < 0.05, (**) *p* < 0.01 (*n* = 4).

Metabolites **2**, **3**, and **4**, three di-desmethylarctigenins, showed IC_50_ values of 7.9 × 10^−4^ M, 1.9 × 10^−3^ M, and 2.5 × 10^−3^ M, respectively. The IC_50_ values of the end metabolite, (−)-DHENL (**1**) was 1.6 × 10^−3^ M. Di-desmethylarctigenins showed rather strong binding affinities compared to mono-desmethylarctigenins. Similarly, in isoflavones, the demethylated metabolites showed stronger estrogen receptor affinity [22]. Remarkable changes in estrogen receptor affinity were observed between daidzein, genistein and their methylated compounds, formononetin and biochanin A.

It has been reported that a phenolic hydroxyl group is a prominent substructural feature for binding to ER and that the distance between two hydroxyl groups is an important factor [23,24]. However, due to the demethylation position, the distances between hydroxyl groups become different. Moreover, further dehydroxylation influences the number of hydroxyl groups and distances because a *para* hydroxyl group which has a vincinal hydroxyl group can be degraded [7].

Arctigenin (**8**) and its metabolites **1**–**7** showed relatively weak ligand binding affinities. However, it becomes clear that the structural differences between the series of these compounds affect the ligand binding affinities to ERalpha.

## 3. Experimental

### 3.1. Chemicals

(−)-Arctigenin (**8**) was isolated from the seeds of *A. lappa* and its metabolites **1**–**7** were prepared by anaerobic incubation of (−)-arctigenin (**8**) as previously reported [20]. 17β-Estradiol was purchased from Calbiochem Co. (Darmstadt, Germany).

### 3.2. Ligand Binding Screen

An estrogen-R (α) competitor screening kit was purchased from Wako Chemicals (Osaka, Japan). The assay depends on the competition between the samples applied in different concentrations and the labeled estrogen mixture. The amount of the ligand that binds to the ERalpha coated on the microplate well is determined by the dynamic equilibrium among all the ligand concentrations in the mixture, the difference of their binding affinities to the receptor, and the incubation time. Therefore, the reduction in fluorescence intensities from the labeled estrogen retained is an indication of the affinity of the added compounds to the estrogen receptor. (−)-Arctigenin (**8**) and its metabolites were tested in 10^−5^, 10^−4^, 5 × 10^−4^, and 5 × 10^−3^ M concentrations. Estradiol was used as a positive control and a labeled estrogen mixture was used as a negative control. The results were calculated as percentages of the negative control.

### 3.3. Statistical Analysis

Each set of experiments was repeated at least three times. Values are expressed as mean ± S.E.M. One-way analysis of variance followed by Dunnett’s test was used for statistical analysis. The IC_50_ was calculated using a non-linear regression analysis (for one site competition).

## 4. Conclusions

In this study, we examined the binding affinities of (−)-arctigenin and its seven demethylated metabolites by *E. limosum* ARC-2 to ERalpha. Though ligand binding affinities were weak compared to estradiol and estrogenic isoflavones, the result showed a tendency that demethylated metabolites are stronger binding ligands to the receptor.
